# Genetic Landscape of a Cohort of 120 Patients with Diminished Ovarian Reserve: Correlation with Infertility

**DOI:** 10.3390/ijms252211915

**Published:** 2024-11-06

**Authors:** Imène Lafraoui, Abdelkader Heddar, Adèle Cantalloube, Inès Braham, Maëliss Peigné, Claire Beneteau, Solenne Gricourt, Claire Poirsier, Stéphanie Legrand, Radka Stoeva, Laure Metayer-Amelot, Annina Lobersztajn, Soizic Lebrun, Nicolas Gruchy, Inès Abdennebi, Isabelle Cedrin-Durnerin, Hervé Fernandez, Dominique Luton, Antoine Torre, Léonore Zagdoun, Nicolas Chevalier, Mohamed Khrouf, Khaled Mahmoud, Sylvie Epelboin, Sophie Catteau-Jonard, Micheline Misrahi

**Affiliations:** 1 Unité de Génétique Moléculaire des Maladies Métaboliques et de la Reproduction, Hôpital Bicêtre, Faculté de Médecine Paris Saclay, INSERM U1193, 94275 Le Kremlin-Bicêtre, France; gafaimen@outlook.fr (I.L.); abdelkader.heddar@gmail.com (A.H.); 2 Laboratoire de Biologie Moléculaire National de Référence-LBMR Pour les Infertilités Génétiques Chez la Femme et l’Homme, Hôpitaux Universitaires Paris Saclay, 94275 Le Kremlin Bicêtre, France; 3 Service de Gynécologie-Obstétrique, Hôpital Tenon, Hôpitaux Universitaires Paris Centre, 75014 Paris, France; adele.cantalloube@aphp.fr (A.C.); solenne.gricourt@aphp.fr (S.G.); sylvie.epelboin@aphp.fr (S.E.); 4 Service d’Endocrinologie, Diabétologie et Médecine de la Reproduction, CHU de Nice, 06000 Nice, France; braham.i@chu-nice.fr (I.B.); chevalier.n@chu-nice.fr (N.C.); 5 Service de Médecine de la Reproduction et Préservation de la Fertilité, Hôpital Jean-Verdier, Université Sorbonne Paris Nord, 93430 Bondy, France; maeliss.peigne@aphp.fr (M.P.); cedrinisabelle@gmail.com (I.C.-D.); 6 Service de Génétique Médicale, CHU de Nantes, 44000 Nantes, France; claire.beneteau@chu-bordeaux.fr; 7 Departement de Genetique, Centre Hospitalier Universitaire de Reims, 51092 Reims, France; cpoirsier@chu-reims.fr; 8 Centre de Fertilité, Clinique de l’Atlantique, 17138 La Rochelle, France; stelegrand33@gmail.com; 9 Laboratoire de Génétique Médicale et Cytogénétique, CH Le Mans, 72037 Le Mans, France; rstoeva@ch-lemans.fr; 10 Service d’Endocrinologie et Médecine de la Reproduction, CH Le Mans, 72037 Le Mans, France; lmetayeramelot@ch-lemans.fr; 11 Centre de la Fertilité—Paris Est, Nogent sur Marne, 94130 Nogent-sur-Marne, France; dr.lobersztajn@centre-de-la-fertilite.com; 12 Service de Génétique, FHU GenOMedS, CHRU de Tours, 37000 Tours, France; s.lebrun@chu-tours.fr; 13 EA 7450 BioTARGen, FHU G4 Genomics, Service de Génétique Clinique, Departement de Genetique, CHU Côte de Nacre, Université de Caen Normandie UNICAEN, 14000 Caen, France; gruchy-n@chu-caen.fr; 14 Centre d’Aide Médicale à la Procréation, Centre Hospitalier Intercommunal de Créteil, 94000 Créteil, France; ines.abdennebi@chicreteil.fr; 15 Service de Gynécologie-Obstétrique, Hôpital Bicêtre, Université Paris Saclay, 94270 Le Kremlin-Bicêtre, France; carfbuffets@gmail.com (H.F.); dominique.luton@aphp.fr (D.L.); 16 Centre d’Assistance Médicale à la Procréation Clinico-Biologique, Centre Hospitalier Sud Francilien Corbeil-Essonnes, 91100 Corbeil-Essonnes, France; antoine.torre@chsf.fr; 17 Service de Diabétologie et Endocrinologie, Centre Hospitalier de Mont de Marsan et Pays des Sources, 40024 Mont de Marsan, France; leonore.zagdoun@ch-mdm.fr; 18 Centre FERTILLIA de Médecine de la Reproduction-Clinique la Rose, Tunis 1053, Tunisia; moh.khrouf@gmail.com (M.K.); drkhaledmahmoud2@gmail.com (K.M.); 19 Service de Gynécologie Endocrinienne, CHU de Lille, Hôpital Jeanne-de-Flandre, 59000 Lille, France; sophie.catteau@chu-lille.fr

**Keywords:** diminished ovarian reserve, primary ovarian insufficiency, cohort, next-generation sequencing, gene, DNA repair, pregnancy, fertility, genetic counseling

## Abstract

Diminished ovarian reserve (DOR) and primary ovarian insufficiency (POI) are major causes of female infertility. We recently found a monogenic etiology in 29.3% of POI, leading to personalized medicine. The genetic landscape of DOR is unknown. A prospective study (2018–2023) of an international cohort of 120 patients with unexplained DOR was performed using a large custom targeted next-generation sequencing panel including all known POI-causing genes. The diagnostic yield, based on the American College of Medical Genetics, was 24, 2%. Genes belong to different pathways: metabolism and mitochondria (29.7%), follicular growth (24.3%), DNA repair/meiosis (18.9%), aging (16.2%), ovarian development (8.1%), and autophagy (2.7%). Five genes were recurrently found: *LMNA*, *ERCC6*, *SOX8*, *POLG*, and *BMPR1B*. Six genes identified in single families with POI were involved in DOR, *GNAS*, *TGFBR3*, *XPNPEP2*, *EXO1*, *BNC1*, *ATG*, highlighting their role in maintaining ovarian reserve. In our cohort, 26 pregnancies were recorded, but no pregnancy was observed when meiosis/DNA repair genes were involved, suggesting severely impaired oocyte quality. Additional studies should confirm these preliminary results. This study with a large NGS panel defines the genetic landscape of a large cohort of DOR. It supports routine genetic diagnosis. Genetics could be a biomarker predicting infertility and progression to POI.

## 1. Introduction

Infertility is a public health problem with a lifetime prevalence in 17.5% of couples in developed countries [1]. Approximately 25% of cases are attributable to ovarian dysfunction [2,3]. Several syndromes can cause this condition, in particular primary ovarian insufficiency (POI) and diminished ovarian reserve (DOR). They are public health problems. POI is defined as the complete cessation of ovarian function before the age of 40 years with amenorrhea or oligomenorrhea ≥4 months and hypergonadotropic hypogonadism with follicle-stimulating hormone (FSH ≥ 25 IU/L) [4]. The prevalence of POI is 1–3.7% [5,6]. Premature DOR is defined as present in women under 35 years with Anti-Mullerian hormone (AMH) plasma levels ≤1.2 ng/L and/or an antral follicle count (AFC) at ultrasonography (US) ≤ 5 [7]. Patients with DOR correspond to 10% of women undergoing assisted reproductive technology (ART), in particular in vitro fertilization (IVF) [8]. However, there are no clinical or biological criteria predicting the response to ovarian stimulation and the success of ART. AMH is an indirect parameter of the ovarian reserve (OR) [5] and, together with AFC, is a quantitative parameter that does not provide information about the quality of the OR and thus the fertility prognosis and success of IVF procedures [7]. The quality of the OR is closely linked to the age of the patient, which is a main factor and the only predictor of IVF success before any attempt [7]. There is no biomarker up to now to assess the quality of the ovarian reserve [7]. The quality of oocytes seems to be correlated with IVF success. Novel POSEIDON (**P**atient-**O**riented **S**trategies **E**ncompassing **I**ndividualize**D** **O**ocyte **N**umber) criteria for low-prognosis patients undergoing ART have been defined [7] (www.groupposeidon.com). To identify patients with a low prognosis in ART, this classification into four groups relies on the age of the patients, quantitative ovarian reserve markers (AMH, AFC, or both), ovarian sensitivity to gonadotropin and the number of oocytes retrieved in previous cycles of conventional ovarian stimulation in cases where this information is available [7]. This suggests the existence of additional factors to oocyte quantity, possibly related to the cause of DOR, that influence the success rate of IVF [9]. In addition, it is difficult to predict which patients with DOR will develop POI in the long term.

Apart from age, known causes of DOR include iatrogenic causes (chemotherapy, radiotherapy, ovarian surgery), viral infections and autoimmune disorders [8,10]. Other causes of DOR including genetic factors are still poorly understood. In a large international cohort of patients with unexplained POI, next-generation sequencing (NGS) using our custom targeted NGS-POI panel comprising all known responsible genes (88) resulted in a high diagnostic yield of 29.3%. This high yield should lead to a routine clinical diagnosis and to personalized knowledge-based medicine [11]. A 23.5% positive genetic diagnosis using NGS has also been subsequently confirmed in a large Chinese cohort of patients with POI with a very similar gene panel (95) [12].

Unlike POI, no NGS studies of large cohorts of DOR have been performed to date to identify the genetic causes of DOR. Only a few studies have been performed, often including isolated families or small cohorts, involving a limited number of genes [13,14,15,16,17,18]. Screening for *FMR1* premutation is occasionally performed but there are conflicting data regarding the association between the *FMR1* premutation and DOR [19,20,21]. The prevalence of genetic causes is therefore unknown, as is the benefit of a genetic diagnosis of DOR in clinical practice.

Here, a large NGS study was performed using our targeted panel of 88 POI-causing genes, in an international cohort of 120 patients with unexplained DOR. Our working hypothesis is that POI and DOR are genetically linked. DOR could be, in some cases, an early stage of POI sharing identical causes. We sought to determine if there is a genetic cause of DOR, if there is a genetic link between DOR and POI and if a genetic screen would be useful in clinical practice for diagnosis and for predicting the occurrence of pregnancies.

## 2. Results

### 2.1. Cohort Characteristics

**Figure 1:**
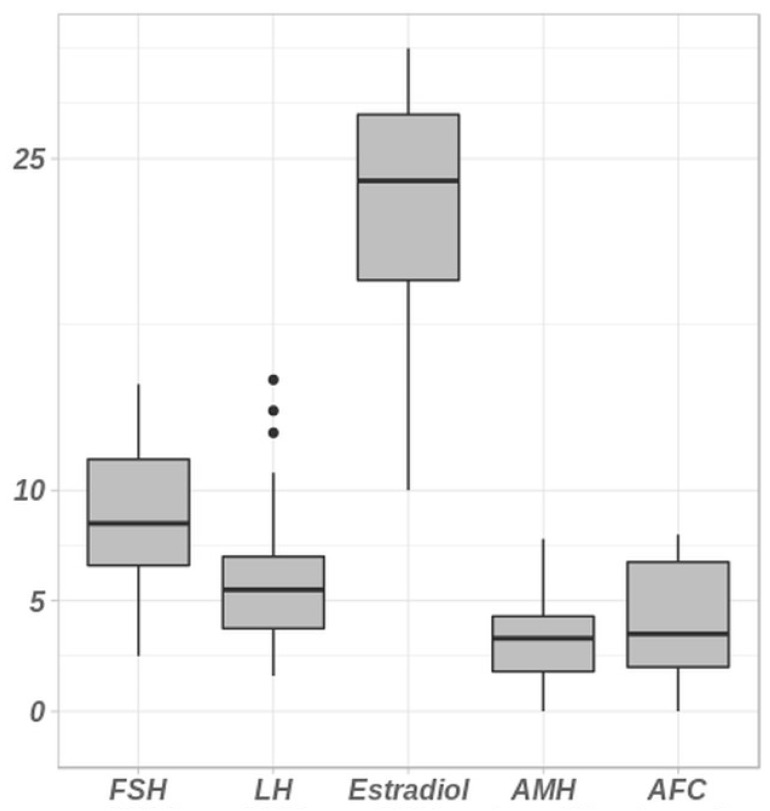
Hormonal assays and antral follicle counts in the cohort of patients with diminished ovarian reserve. Hormonal assays (FSH, LH, Estradiol and AMH) together with the antral follicle count (AFC) in patients. The Y scale unit is variable according to each parameter represented on the *X*-axis. Normal range: AMH (7–20.7 pmol/L); respectively, ranges for follicular, ovulatory, luteal phases and menopause are the following: FSH (IU/L): (2.9–12), (6.3–24), (1.5–7), (17–95); LH: (IU/L) (1.5–8), (9.6–80), (0.2–6.5), (8–33); Estradiol (ng/L): (19.5–144.2), (63.9–356.7), (55.8–214.2), (≤32.2).

The clinical descriptions of the patients recruited in this study and studied between 2018 and 2023 in our National Infertility Genetics Reference Laboratory are summarized in Table 1 and Figure 1 (see also Section 4). Patients with DOR fulfill the POSEIDON criteria group 3 [7]: patients ≤ 35 years with AFC < 5 and/or AMH < 1.2 ng/mL (8.6 pmol/L). The international cohort of unexplained DOR included 120 patients mainly of European ancestry (75.5%) (Table 1). Patients also originated from North-Africa (20%), Africa (2%) or Asia (2.5%). The mean age of the patients was 30.8 (16–35) years. AMH levels ranged from 0.03 to 1.2 ng/L with an average of 0.46 ng/L and AFC ranged from 0 to 8 with an average of 4.17. Twenty-six pregnancies were recorded in the whole cohort (20.8%), twenty-five spontaneous and one with IVF.

### 2.2. Molecular Findings

#### 2.2.1. Positive Genetic Diagnosis in DOR and Different Pathways Implicated

**Figure 2:**
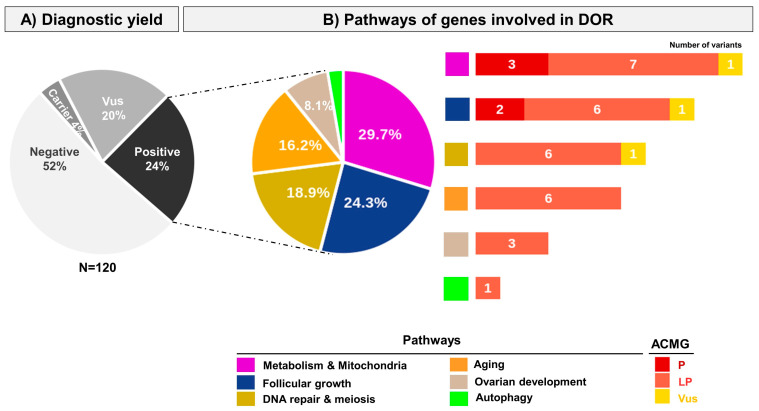
Genetic studies of a cohort of patients with DOR. Patients were studied by a custom-made targeted NGS comprising 88 genes. (**A**) Diagnostic yield according to ACMG criteria: variants are classified according to the American College of Medical Genetics (ACMG) guidelines. N = 120: the whole cohort comprises 120 patients with DOR. Vus: variant of unknown significance. Carrier: patients harboring a heterozygous pathogenic variant in a known autosomal recessive POI gene. Positive: the diagnostic yield corresponds to patients carrying pathogenic (P) or likely pathogenic (LP) variants and is 24%. (**B**) Pathways of genes involved in DOR. The different pathways are indicated with different colors. The pie chart represents the proportion of patients with P or LP variants in a specific pathway: Mitochondria and Metabolism (29.7%), Follicular growth (24.3%), DNA repair and meiosis (18.9%), Aging (16.2%), Ovarian development (8.1%), Autophagy (2.7%). The histograms show the number and type of variants detected in each pathway.

In the whole cohort, our targeted NGS-POI panel identified 64 variants in 56 patients (out of 120) (Figure 2, Table 2 and Table 3). Some variants identified in the cohort of patients with DOR are pathogenic (P) or likely pathogenic (LP) according to the American College of Medical Genetics (ACMG) and are found on two or one alleles, consistent with a known recessive or dominant inheritance of the gene. They are causal (Table 2). Other variants are pathogenic variants but involve a single allele in genes known to have recessive inheritance in POI, or are variants of unknown significance. They are not causal (Table 3). Using ACMG criteria, 31 P/LP variants were identified in 29 patients. The diagnostic performance of our NGS study was of 24.2% (29/120) for the whole cohort and 26.4% (24/91) for European patients (n = 91). No P/LP copy number variations (CNVs) were detected.

We previously found a diagnostic positivity of 29.3% in our cohort of patients with POI with the custom-made NGS panel containing all known genes responsible for POI [11]. In this study, with the same NGS-POI panel, a 24.2% positivity is found for the genetic diagnosis of patients with DOR. This shows the similarity of the genes involved in both syndromes and their genetic proximity. It also confirms that DOR in some cases can be early forms of POI [11].

The genes displaying P/LP variants are involved in different pathways (Figure 2): 29.7% are involved in metabolism and mitochondrial functions, 24.3% are involved in follicular growth, 18.9% are involved in meiosis/DNA repair, 16.2% are involved in nuclear lamina (aging), 8.1% are involved in ovarian development and 2.7% are involved in autophagy (Figure 2). This result markedly contrasts with the major involvement of the meiosis/DNA repair gene family in our previous cohort of patients with POI (37.4%) [11].

#### 2.2.2. Confirmation of the Causal Role of Genes in Ovarian Function

Some genes were previously identified in single patients or single families with POI. Their identification in our cohort of patients with DOR confirms the role of these genes in ovarian functions and highlights their role in the maintenance of the OR.

A pathogenic variant, NM_080425.3:c.2524C>T:p.Arg842Cys, was detected in GNAS (Table 2) in one patient with DOR and obesity. The Gs alpha gene (GNAS) is expressed in different tissues and involved in numerous signaling pathways. Loss of function mutations of GNAS cause pseudohypoparathyroidism [5]. Menstrual irregularities with ovarian dysfunction have been described in women with pseudoparathyroidism in one study [22]. Interestingly, the Arg842Cys variant was recently reported in a patient presenting with isolated obesity without the classical signs of pseudo hypoparathyroidism or Albright’s syndrome, related to GNAS alteration [23]. Functional characterization demonstrated a functional impact of this variant on cyclic AMP production in vitro [23].

In one patient, a truncated variant NM_003243:c.2059C>T p.Arg687Ter in *TGFBR3* was identified (Table 2). This gene encodes a receptor of the TGF-β ligand family [5,24]. TGFBR3 has been previously suggested to be involved in POI through the discovery of single polymorphisms in only two patients [25,26].

In this study, the second truncated variant of *XPNPEP2* was identified, NM_003399.5: c.235-1G>C p.?, (Table 2). This gene is implicated in the metabolism of peptides. A balanced (X;12) translocation disrupting *XPNPEP2* was initially found in a patient with POI and her affected mother [27]. We have recently described the first heterozygous frameshift deletion in exon 2 of *XPNPEP2* in a patient with POI [11]. This first LP variant in a patient with DOR confirms the role of this gene in human female infertility and highlights its role in the maintenance of the OR.

In another patient, a truncated variant NM_130398.4:c.2485G>T p.Glu829Ter in *EXO1* was identified (Table 2). This gene encodes an exonuclease with a crucial role in meiotic recombination. Recent studies have found that EXO1 also promoted crossover resolution, emphasizing the crucial role of EXO1 in meiosis [28,29]. A single heterozygous variant in *EXO1* had been described in a Chinese patient with POI and the homologous p.Thr52Ser EXO1 variant impaired the meiotic process of budding yeast. This variant also impaired the efficiency of homologous recombination repair for DNA double-stranded breaks in human cells [30].

A novel pathogenic splice variant of *BNC1* NM_001717.4:c.436-2A>G p.? was identified in this study (Table 2). BNC1 was recently shown to play key roles in OR [31,32]. Deficiency of BNC1 results in premature follicular activation and excessive follicular atresia [31].

A novel splice variant of *ATG7* (Table 2), a gene implicated in autophagy, was identified in this study. *ATG7* has been previously involved in two isolated patients with POI [11,33].

#### 2.2.3. Genes Recurrently Involved in the Cohort of Patients with DOR

**Figure 3:**
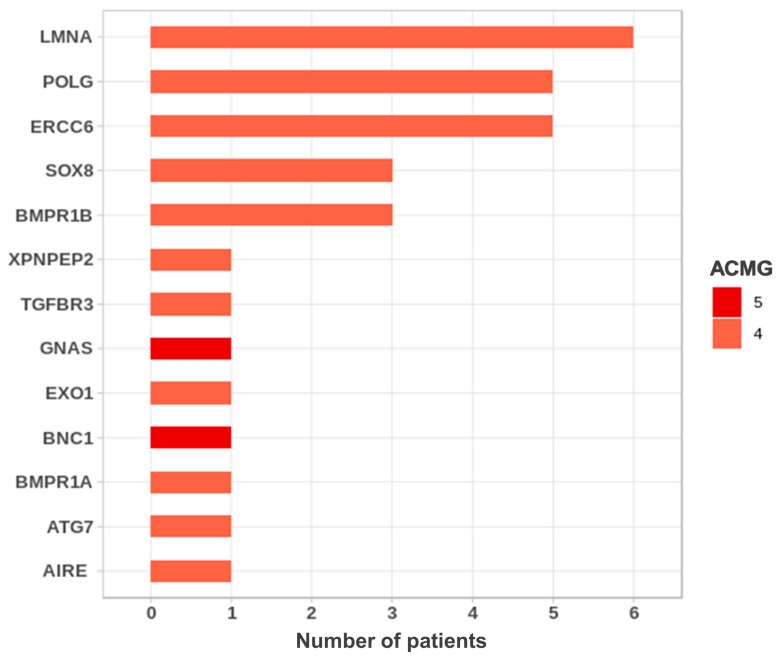
Genes recurrently involved in the cohort of DOR. Variants are classified according to ACMG criteria. Only pathogenic (class 5) and likely pathogenic (class 4) variants are considered for genetic diagnosis as recommended by the ACMG. The number of patients carrying a class 5 or class 4 variant of each gene is indicated.

We detected five genes with variants found in at least three patients. These are *LMNA*, *ERCC6*, *POLG*, *SOX8* and *BMPR1B* (Figure 3).

Variants in the *LMNA* gene were most frequently observed in the DOR cohort (Figure 3). This gene encodes Lamins A and C, the nuclear intermediate filament proteins that play a major architectural role in the cell nucleus [34]. *LMNA* is involved in aging [34]. Pathogenic variants in this gene, which are most often heterozygous, cause laminopathies that include a large phenotypic spectrum of rare diseases, in particular muscular dystrophy, familial lipodystrophy, progeria, atypical progeroid syndromes, Hutchinson–Gilford’s disease with accelerated aging, mandibuloacral dysplasia, and atypical Werner syndrome with hypogonadism [34]. Three patients presenting POI with dilated cardiomyopathy have been reported [35,36]. Six different P/LP variants in six unrelated patients with apparently isolated DOR were found in this study (see Table 2). In a 35-year-old patient, the NM_170707.4:c.1633C>T p.Arg545Cys variant located in the C-terminal part of the protein was found. This variant has been previously reported in a patient with autosomal Emery–Dreifus muscular dystrophy [37]. It is reported in the Clinvar database in patients with cardiomyopathy, muscular dystrophy or Charcot–Marie–Tooth disease. Two independent functional studies performed on the patient’s myoblasts demonstrated that the Arg545Cys variant leads to pleiotropic cellular defects, in particular nuclear structural defects [37,38]. Two other *LMNA* variants, NM_170707.4:c.647G>A p.Arg216His and NM_170707.4:c.1201C>T p.Arg401Cys, were identified in two patients aged 29 and 34 years (Table 2). These variants have been shown to produce several nuclear anomalies [39]. Both variants were also reported in ClinVar in patients with cardiomyopathy, muscular dystrophy or Charcot–Marie–Tooth disease. We recommended retro-phenotyping in patients and their families.

The second gene recurrently mutated is *ERCC6,* with causal variants identified in five patients with DOR. *ERCC6* is a transcription-coupled DNA repair gene causing POI with a dominant mode of transmission [40]. Interestingly, the same variant NM_000124.4:c.1996C>T p.Arg666Cys was identified in four unrelated European patients with DOR (Table 2). This variant was also previously found in two unrelated Caucasian patients with POI [11]. In another 32 year-old patient, a novel *ERCC6* P variant was found, NM_000124.4:c.2924G>T, involving the last nucleotide of exon 16, yielding p.Arg975Leu. This variant is highly predicted to alter the splicing of *ERCC6* intron 16.

The third gene recurrently mutated is *POLG*, which is involved in mitochondrial function. *POLG* encodes a DNA polymerase g, a catalytic subunit that is responsible for the replication of the mitochondrial genome. Mutations in *POLG* yield impaired mitochondrial DNA replication and maintenance [41]. Syndromic POI with neurosensory symptoms is associated with mutations of this gene [5]. Two patients in this study harbored the variant NM_001126131.2:c.1550G>T yielding p.Gly517Val and two patients harbored the variant NM_001126131.2:c.2492A>G yielding p.Tyr831Cys that was previously reported in patients with Parkinsonism or ophthalmoplegia, belonging to the phenotypic spectrum of *POLG* [42]. One patient had syndromic DOR associated with an essential tremor (see below). Two isolated cases of DOR at diagnosis were also associated with *POLG* P/LP variants, as described for isolated POI [43]. Our findings, as well as previous observations, highlight the major roles of mitochondria in oocyte energy homeostasis and in the OR [41,44].

Two variants of *SOX8*, a gene with a crucial role in ovarian development, were detected in three unrelated patients (Table 2). These variants were previously described in patients with a wide spectrum of human reproductive disorders [45] and in our cohort of patients with POI [11].

Three patients had variants in *BMPR1B* (Table 2). This gene encodes a transmembrane receptor, a member of the bone morphogenetic protein (BMP) receptor family expressed in lungs and ovaries [24,46]. It is involved in folliculogenesis [24]. One variant was identified in a patient and her mother and the second variant was identified in an unrelated patient (Table 2).

#### 2.2.4. Genes Involved in Syndromic DOR

In 16 patients, variants were found in genes known to be involved in a Mendelian disorder not only limited to an ovarian phenotype or POI including the genes *LMNA*, *POLG*, *GNAS*, *AIRE*, *BMPR1A* and *BMPR1B*. In some patients, somatic extra ovarian features related to the phenotypic spectrum of the gene were detected. One out of the five (1/5) patients carrying a pathogenic variant of *POLG* presented an essential tremor that could fit with the phenotypic spectrum of *POLG*. The patient with a GNAS variant had syndromic DOR with major obesity (BMI: 44) as described recently [23] and a thyroid goiter. Of the six patients with variants in *LMNA*, two patients had extra ovarian symptoms, one presented nephroblastoma and deafness, and the other presented early breast cancer at the age of 20 years. However, in the other patients no extra ovarian symptoms could be identified.

For all patients with isolated DOR at the time of the study, but with variants in syndromic genes, like *LMNA*, *POLG*, *BMPR1A* or *BMPR1B*, comprehensive phenotyping of patients and presymptomatic carriers in the family is recommended to detect possible associated symptoms, with appropriate follow-up in a multidisciplinary team if necessary.

Our observation also supports a comprehensive initial evaluation of patients with DOR, looking for the involvement of other organs. The presence of a syndromic DOR can immediately direct the genetic diagnosis towards a specific genetic cause.

#### 2.2.5. Co-Occurrence of Variants in Different Genes

In seven patients, the co-occurrence of variants in different genes was found. In six cases, one mutated gene was reported in patients with POI and dominant transmission inheritance of the gene, suggesting that the alteration of these genes alone is sufficient to cause the phenotype [5,40,45]. This is the case for the heterozygous mutation of *ERCC6*, *POLG* and *SOX8* [5,40,45]. However, one patient had P/LP variants in both *ERCC6* and *POLG*. In this case, the contribution of both genes in the syndrome cannot be excluded. But this single patient is not sufficient to make conclusions for a digenic inheritance in DOR.

#### 2.2.6. Heterozygous Variants in Known Recessive Genes

In four patients, a single PV/LP variant was found in autosomal recessive genes known to cause POI (Table 3). This most likely corresponds to a carrier status (Figure 2) or may indicate a new mode of inheritance for these variants. Supplementary cases are needed to make a definitive conclusion.

#### 2.2.7. Correlation Between the Genetic Cause and the Occurrence of Pregnancies

In the whole cohort, twenty-six pregnancies were recorded and, in patients with an established genetic diagnostic, thirteen pregnancies. In almost all cases, there was one pregnancy per patient, with the exception of two patients with an *LMNA* defect (two pregnancies for two patients) and the patient with a *POLG* defect (two pregnancies in one patient).

**Figure 4:**
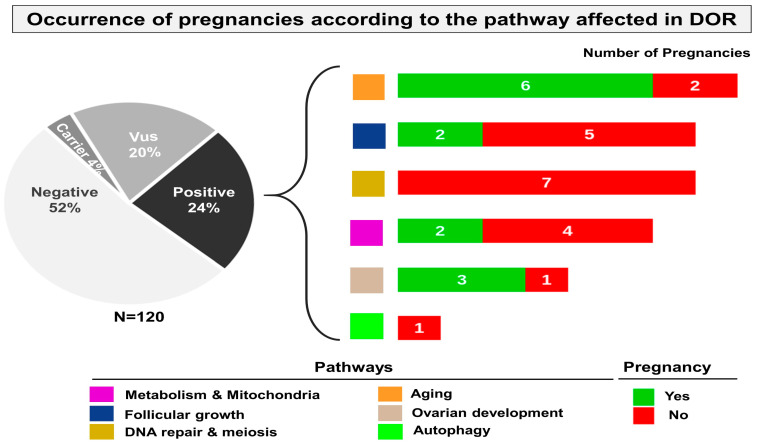
The histograms show the number of pregnancies recorded in patients with alterations in each genetic pathway. Each pathway is represented by a color code. The occurrence of pregnancies is represented in green with the number of pregnancies indicated for each pathway altered. The number of patients without a pregnancy is shown in red for each pathway altered. Thirteen pregnancies were recorded in patients with an established genetic diagnostic with, in almost all cases, one pregnancy per patient except for two patients with an *LMNA* defect (two pregnancies for two patients) and the patient with a *POLG* defect (two pregnancies in one patient).

However, no pregnancy occurred in patients with DOR with molecular alterations in DNA repair genes, suggesting a poor prognosis for this gene family that can significantly impair oocyte quality (Figure 4). Patients with mutations of other gene families seem to have a better prognosis of fertility, i.e., genes involved in follicular growth, metabolism and mitochondrial functions, aging and ovarian development. Only one patient presented a likely pathogenic variant in a gene involved in autophagy, which does not allow any conclusions to be drawn.

## 3. Discussion

Here, a custom-made NGS-POI panel of 88 POI-causing genes was used to study a large cohort of 120 patients with DOR in order to determine the possibility of a genetic diagnosis in routine clinical practice, to define the genetic architecture of DOR and to define genetic links with POI. This analysis could help determine which patients with DOR may progress to POI. The genetic study would allow personalized patient care and an assessment of fertility prognosis.

An important finding of our study is that the diagnostic yield of this NGS strategy is 24%. This result supports a routine genetic diagnosis for all patients with unexplained DOR.

Few genetic studies were previously reported in patients with DOR, including single cases/families or small cohorts with a limited number of genes studied [13,18,47,48]. These studies did not examine systematically the yield of the genetic diagnosis in DOR, the different pathways involved and the prevalence of each gene/pathway in a large cohort of DOR. They did not provide arguments for the use of genetics in routine clinical practice for the diagnosis of DOR and did not study the link between a genetic cause and the occurrence of pregnancies.

We show here that the genetic landscape of DOR is heterogeneous. Four main pathways are involved: mitochondrial function and metabolism (29.7%), follicular growth (24.3%), DNA repair (18.9%) and aging (16.2%). These findings highlight the crucial role of these different pathways in the maintenance of the OR.

There is an increasing consensus to associate pathways involving mitochondrial dysfunction in oocyte energy homeostasis and maintenance of the OR [44]. Mitochondrial dysfunction yields oxidative stress and perturbs oocyte–cumulus cross-talk [44].

Furthermore, genes involved in follicular growth, notably those of the TGF-b/Smads signaling pathway, have crucial roles in the regulation of follicular development. This superfamily includes several proteins, i.e., GDF9, BMP15, BMPR2 and inhibin, involved in the development of ovarian follicles [5,24]. Abnormalities in either process of the transduction pathway can result in impaired follicular recruitment, the inhibition of follicular growth and development, and accelerated follicular atresia [49]. Interestingly, in addition to *BMPR1A*, *BMPR1B* has been implicated in POI in humans [11,50] and also in mice [51]. Here, evidence is provided for the involvement of TGFBR3, another receptor of the TGF-b/Smads pathway, in ovarian function and in the maintenance of the OR. Our data also support a role for *BNC1* in the ovary [11,32] and in the maintenance of the OR. Pharmacologic inhibition of YAP signaling or ferroptosis significantly rescues Bnc1 mutation-induced POI [31] and may correspond to potential novel therapeutic targets.

Aging is also a crucial pathway involved in DOR. Lamin A is involved in the aging process. Variants in *LMNA* have been described previously in only three patients with apparently isolated POI [11,12]. Remarkably, this gene is the most recurrently mutated in our cohort and these data confirm its involvement in the maintenance of the OR. It has been proposed that mutations which cause chromosomal instability and premature aging also lead to accelerated follicular loss in the ovary and reduced fertility [5]. Another group suggested that Lamin A acts as a sensor of intrinsic and environmental cellular stress via the transient accumulation of prelamin A, which triggers stress response mechanisms. Exacerbation of Lamin A sensor activity due to stable and high levels of prelamin A contributes to the onset of a permanent stress response condition, which triggers accelerated aging [34].

Finally, the DNA repair/meiosis gene family is also involved in DOR, although in a much lower proportion than for POI, respectively, 18.2% in DOR and 37.4% in POI [11]. This could be explained by the fact that pathogenic variants in these genes (for instance *STAG3*, *MCM8* or *PSMC3IP*) result most often in severe changes in the maintenance of the ovarian reserve and cause a rapid loss of ovarian function, resulting in POI and primary amenorrhea [5].

Inactivation of *EXO1* in mice resulted in infertility, ovarian dysgenesis, and POI, which was attributed to a dynamic loss of chromosomes chiasmata during the first meiotic prophase, indicating its pivotal role in oogenesis [29]. This first variant of *EXO1* found in DOR confirms the involvement of *EXO1* in female infertility in humans and in the maintenance of the OR. Increased predisposition to cancer has been reported in patients carrying variants in *EXO1* [29] but not confirmed. However, no history of tumors was reported in the patient previously described with POI and in our patient with DOR and *EXO1* LP variant.

Autophagy is a cytolytic metabolic pathway that helps regulate the various stages of oocyte death and follicular atresia during development. In high-stress conditions, autophagy inhibits apoptotic signals in ovarian oocytes and/or granulosa cells and promotes oocyte and follicle survival [33]. We confirm here the role of *ATG7* in the maintenance of the OR.

Consistently, all genes involved in DOR here have been described in POI with secondary amenorrhea or oligomenorrhea, suggesting, in the latter patients, the existence of a residual ovarian reserve.

Our results support the existence of a continuum between DOR and POI. Genetic studies could identify patients at risk of developing POI, with early diagnosis and appropriate care for the patient and their family. Fertility preservation may be offered to these patients and their affected siblings.

Interestingly, we show here that DOR can be the only apparent symptom of a more complex disease that may include other extra-ovarian features and therefore requires adapted genetic counseling and medical care. This is the case, for example, when genes such as *LMNA*, *AIRE*, *GNAS*, *POLG*, *BMPR1A* or *EXO1* are involved. This is consistent with our previous findings in patients with POI [11]. In our previous POI cohort, 10.4% of patients initially referred to as having isolated POI were found to have pathogenic variants of genes causing syndromic POI. Other organ defects were discovered after the re-evaluation of the patients. Reverse phenotyping could result in the identification of other organ defects linked to mutations in the same gene. The existence of potential defects in other organs requires a complete evaluation of patients, appropriate genetic counseling and personalized care of patients and families either for the prevention/treatment of other associated organ defects. However, in some cases, no other symptom can be identified. This can be explained by the phenotypic or penetrance variability, or even by the fact that such extra ovarian symptoms are underestimated or not yet expressed or apparent in these patients. The existence of potential anomalies in other organs requires a comprehensive evaluation of patients and most often the long-term follow-up of patients and their families.

The identification of reliable markers of OR, but also of fertility biomarkers, is a major challenge in the field of reproduction. Predicting spontaneous pregnancies or ART/IVF success criteria in patients with DOR remains a challenge in clinical practice. DOR is currently defined on the basis of quantitative criteria: plasma AMH levels and AFC. However, AMH does not provide any direct indication of the ovarian reserve, which consists only of primordial follicles devoid of granulosa cells, which are the only ones that secrete AMH. AMH assays may be negative even though there is an ovarian reserve without sufficient follicle growth in the ovaries to secrete AMH that is detectable in the peripheral blood [5]. AFC is dependent on the practitioner that performs the examination. In addition, there is much controversy over the relationship between oocyte quantity and quality [9,52,53]. It is unclear whether an abnormally low OR is necessarily accompanied by a concomitant reduction in oocyte quality [9,53]. This discrepancy may explain the discordant results of numerous studies between AMH levels and pregnancy outcomes in patients with DOR [9,52,53].

We hypothesized that genetics could provide a reliable biomarker of patients’ fertility prognosis through the precise identification of the molecular signatures involved. We thus compared the occurrence of pregnancy in the patients according to the genetic variants identified in this study. Thirteen pregnancies were observed in the patients with an established genetic diagnosis. However, no pregnancies were achieved in patients with molecular defects in meiosis/DNA repair genes, suggesting unfavorable poor prognosis when this gene family is involved. Indeed, altered oocyte quality through altered meiosis or altered maturation is expected. On the other hand, patients with an alteration of genes involved in metabolism or mitochondrial function, in follicular growth or aging have better pregnancy success rates. Additional studies, including larger cohorts, are necessary to confirm our preliminary results and to know whether genetics could provide a fertility prognosis in patients with DOR.

Genetic studies could serve as new early biomarkers to assess the quality of the ovarian reserve in patients with DOR ≤ 35 years and to predict the outcome of ART. Identification of the cause of DOR could highlight an alteration in DNA repair mechanisms and meiotic processes that mainly impact the quality of the OR. There is an urgent need for biomarkers to predict ART success to avoid patient and couple suffering and high costs to society in terms of healthcare expenses. This has implications for the genetic counseling of patients, especially since in industrialized countries a delay in the age of first pregnancy after 30 years of age is common.

## 4. Materials and Methods

### 4.1. Patients

An international cohort of 120 patients with unexplained DOR was screened between 2018 and 2023 in our National Infertility Genetics Reference Laboratory. The patients with DOR fulfill the POSEIDON criteria group 3 [7]: patients ≤ 35 years with AFC < 5 and/or AMH < 1.2 ng/mL (8.6 pmol/L). The mean age of the patients was 30.8 (16–35) years. The 16-year-old patient was studied because of an unrelated associated disease for which hormonal assays were performed, revealing the existence of DOR. Our cohort consists of patients with unexplained DOR, a normal karyotype and the *FMR1* gene. Patients with possible etiologies for their DOR were excluded from the study. The exclusion criteria are as follows: primary ovarian insufficiency: patients with oligomenorrhea, secondary amenorrhea and plasma FSH levels ≥ 20 IU/L; polycystic ovarian syndrome with possible menstrual disorders and infertility but high AMH values; patients with an associated disease that could impact the ovarian reserve: ovarian surgery, chemotherapy, pelvic infections, pelvic irradiation, endometriosis with surgery for ovarian endometrioma.

The following clinical data were recorded in the patient and/or family before the NGS analysis: ethnicity, autoimmune diseases, the presence of extra-ovarian symptoms, such as intellectual disability, cardiac or neurological symptoms, a family history of male or female infertility, together with the occurrence of pregnancies either spontaneously or after IVF. We also requested US findings to assess ovarian and follicular sizes and the number of follicles, as well as complete hormonal assays in particular of FSH, luteinizing hormone (LH), estradiol and AMH plasma levels.

### 4.2. Genetics Studies

Patients were studied in a National Reference Laboratory for infertility genetics. Informed signed consent was obtained from the patients for the identification of a genetic cause of their DOR before the genetic study. Blood was then extracted from peripheral white cells and genomic DNA was extracted from blood samples by standard protocols [11]. A custom-made targeted NGS panel including all known genes involved in POI to date (n = 88) was used as previously described [11,54]. Libraries were prepared using an NEB Next DNA Library Prep Master Mix Set for Illumina (NEB Inc., Ipswich, MA, USA). Enrichment was performed on 200 ng of DNA using a Sure Select XT Reagent Kit (Agilent, Santa Clara, CA, USA). Libraries were subjected to 75 bp of paired-end sequencing on a MiSeq2500 according to the manufacturer’s protocol. Data analysis was performed using an automated bioinformatics pipeline implemented with a local Galaxy instance at Bicêtre Hospital, France [55]. In brief, alignment to the human reference genome 19 (GRCh37) was performed using BWA-MEM 0.7.10. Variant calling was performed using GATK 3.4-46, and single nucleotide variants were annotated using both Annovar 2015-06-17 and Snpeff 4.0. Variants detected were processed using the following bioinformatic filters: (i) variants with a read coverage under 5× and a Q score < 20 were filtered out, and (ii) variations against public and available databases including ExAC and gnomAD databases were filtered using a minor allelic frequency (MAF) of 0.01. Variant classification according to the ACMG-AMP 2015 guideline was performed using InterVar [56] (https://wintervar.wglab.org/ accessed on 13 June 2022), Varsome [57] (https://varsome.com/, accessed on 9 June 2023) and MobiDetails [58] (https://mobidetails.iurc.montp.inserm.fr/MD/, accessed on 31 December 2023). We only considered variants classified as pathogenic or likely pathogenic according to ACMG guidelines [59]. Copy Number Variations (CNV) were studied using an in-house coverage-based pipeline [11].

### 4.3. Ethics Statement

The study was approved by all the institutions involved and by the Agence de Biomedecine (reference number PFS12-002). Written informed consent was received from all participants prior to inclusion in the study.

## 5. Conclusions

Genetic study of a large cohort of patients with unexplained DOR with the custom-made NGS-POI panel yielded a 24% genetic diagnosis, supporting the use of NGS in routine clinical practice. There is a genetic link between POI and DOR, which share common causes. Four genetic pathways explain ~90% of genetic defects in DOR: metabolism and mitochondrial functions (29.7%), follicular growth (24.3%), DNA repair/meiosis (18.9%) and aging (16.2%). DOR can precede POI and regular monitoring of patients should be performed to assess ovarian function. Appropriate genetic counseling should be performed for patients and their families. Fertility preservation may be offered to patients and their affected siblings. Associated symptoms may occur when the gene involved is a syndromic gene or may yield associated comorbidities. Such comorbidities must be detected, prevented and/or treated in a multidisciplinary team. This work shows that personalized genetic counseling and care is possible in DOR based on the identification of the genetic cause. The occurrence of pregnancy appears to be low in patients with alterations in DNA repair genes while fertility prognosis seems to be better when other pathways are involved. Further genetic studies in larger cohorts are needed to confirm this pilot study and to better elucidate the molecular mechanisms underlying DOR. Genetic studies could predict the outcome of ART through the identification of the cause that could impact not only the quantity but also the quality of oocytes.

## Figures and Tables

**Table 1:** Clinical and biological characteristics of the cohort of patients with diminished ovarian reserve.

**Mean Age**	30.8 [16–35]	
**Ethnicity**	**Number of Patients**	**Percentage**
European	91	75.5%
North-African	24	20%
African	2	2%
Asian	3	2.5%
**Initial Clinical Presentation**		
Isolated	113	94%
Syndromic	7	6%
**Pregnancy**		
Yes spontaneously	25	20.8%
1 pregnancy	18	14.4%
2 pregnancies	6	4.8%
3 pregnancies	1	0.8%
Yes with IVF	1	0.8%
No	94	78.4%
**Hormonal Assays**	**Mean**	**Range**
FSH (IU/L)	8.9	[2.52–14.8]
LH (IU/L)	5.69	[1.6–15]
Estradiol (IU/L)	47.19	[10–113]
AMH (ng/L)	0.46	[0.03–1.2]

FSH: follicle-stimulating hormone; LH: luteinizing hormone; AMH: Anti-Mullerian hormone.

**Table 2:** List of pathogenic or likely pathogenic variants identified in the cohort of patients with diminished ovarian reserve.

ID	Ethnicity	Age	Pregnancy	Gene	Variant	Haplotype	ACMG	Pathway
101	European	34	0	*EXO1*	NM_130398.4:c.2485G>T p.(Glu829Ter)	Het	4	DNA Repair and Meiosis
24	European	32	0	*ERCC6*	NM_000124.4:c.2924G>T p.(Arg975Leu)	Het	4	DNA Repair and Meiosis
33	European	26	0	*BNC1*	NM_001717.4:c.436-2A>G p.?	Het	5	Follicular Growth
41	European	30	0	*ERCC6*	NM_000124.4:c.1996C>T p.(Arg666Cys)	Het	4	DNA Repair and Meiosis
				*POLG*	NM_001126131.2:c.1760C>T p.(Pro587Leu)	Het	5	Metabolism and Mitochondria
				*POLG*	NM_001126131.2:c.752C>T p.(Thr251Ile)	Het	4	Metabolism and Mitochondria
42	European	34	0	*ERCC6*	NM_000124.4:c.1996C>T p.(Arg666Cys)	Het	4	DNA Repair and Meiosis
44	European	28	0	*ERCC6*	NM_000124.4:c.1996C>T p.(Arg666Cys)	Het	4	DNA Repair and Meiosis
83	European	32	0	*ERCC6*	NM_000124.4:c.1996C>T p.(Arg666Cys)	Het	4	DNA Repair and Meiosis
77	North-African	35	2	*LMNA*	NM_170707.4:c.1633C>T p.(Arg545Cys)	Het	4	Aging
17	European	29	0	*LMNA*	NM_170707.4:c.647G>A p.(Arg216His)	Het	4	Aging
45	European	32	1	*LMNA*	NM_170707:c.659G>A p.(Arg220His)	Het	4	Aging
12	European	31	0	*LMNA*	NM_170707.4:c.350A>G p.(Lys117Arg)	Het	4	Aging
			0	*POLG*	NM_001126131.2:c.678G>C p.(Gln226His)	Het	3	Metabolism and Mitochondria
20	European	34	2	*LMNA*	NM_170707.4:c.1201C>T p.(Arg401Cys)	Het	4	Aging
70	European	34	1	*LMNA*	NM_170707:c.1718C>T p.(Ser573Leu)	Het	4	Aging
15	European	27	0	*POLG*	NM_001126131.2:c.2209G>C p.(Gly737Arg)	Het	4	Metabolism and Mitochondria
84	European	32	0	*POLG*	NM_001126131.2:c.1550G>T p.(Gly517Val)	Het	4	Metabolism and Mitochondria
38	European	34	0	*POLG*	NM_001126131.2:c.1550G>T p.(Gly517Val)	Het	4	Metabolism and Mitochondria
56	European	30	2	*POLG*	NM_001126131.2:c.2492A>G p.(Tyr831Cys)	Het	4	Metabolism and Mitochondria
99	North-African	34	0	*XPNPEP2*	NM_003399.6:c.235-1G>C p.?	Het	4	Metabolism
1	European	16	0	*GNAS*	NM_080425.3:c.2524C>T p.(Arg842Cys)	Het	5	Follicular Growth
95	European	34	1	*TGFBR3*	NM_003243):c.2059C>T p.(Arg687ter)	Het	4	Follicular Growth
101	European	35	0	*AIRE*	NM_000383:c.601G>T p.(Gly201Ter)/c.586T>A p.(Ser196Thr)	Comp Het	4/3	Follicular Growth
65	European	20	0	*BMPR1A*	NM_004329.3:c.850C>T p.(Arg284Cys)	Het	4	Follicular Growth
61	North-African	34	0	*BMPR1B*	NM_001256793:c.1165A>G p.(Ser389Gly)	Het	4	Follicular Growth
88	European	35	0	*BMPR1B*	NM_001256793.2:c.761G>A p.(Arg254His)	Het	4	Follicular Growth
				*GALT*	NM_000155.4: c.563A>G p.(Gln188Arg)	Het	5	Metabolism and Mitochondria
102	European	33	1	*BMPR1B*	NM_001256793.2:c.761G>A p.(Arg254His)	Het	4	Follicular Growth
				*GALT*	NM_000155.4: c.563A>G p.(Gln188Arg)	Het	5	Metabolism and Mitochondria
46	European	30	2	*SOX8*	NM_014587.5:c.1144G>A p.(Asp382Asn)	Het	4	Ovarian development
48	North-African	34	1	*SOX8*	NM_014587.5:c.1144G>A p.(Asp382Asn)	Het	4	Ovarian development
				*PCCB*	NM_000532.5:c.1220G>T p.(Gly407Val)	Het	4	Metabolism and Mitochondria
57	European	34	0	*SOX8*	NM_014587.5:c.1246G>A p.(Ala416Thr)	Het	4	Ovarian development
			0	*ERCC6*	NM_000124.4:c.2875G>C p.(Val959Leu)	Het	3	DNA Repair and Meiosis
86	North-African	35	0	*ATG7*	NM_006395.3:c.1277C>T p.(Pro426Leu)	Het	4	Autophagy

ACMG: classification of the variant according to the guidelines of the American college of Medical genetics. Het: heterozygous: Comp Het: compound heterozygous. The age in the table refers to the age of onset.

**Table 3:** List of monoallelic pathogenic/likely pathogenic variants in recessive POI genes or variants of unknown significance in the cohort of patients with diminished ovarian reserve.

ID	Ethnicity	Age	Pregnancy	Gene	Variant	Haplotype	ACMG	Pathway
48	European	33	1	*POLG*	NM_001126131:c.1399G>A:p.A467T	Het	4 (Carrier)	Metabolism and Mitochondria
54	European	25	0	*PMM2*	NM_000303.3:c.713G>A:p.Arg238His	Het	4 (Carrier)	Metabolism and Mitochondria
83	Turkish	27	0	*ATM*	NM_000051.4:c.2502dup p.Val835SerfsTer7	Het	4 (Carrier)	DNA Repair and Meiosis
78	North-African	33	0	*ATM*	NM_000051.4:c.8781_8786+2del/c.1810C>T:p.Pro604Ser	Het	4 (Carrier)	DNA Repair and Meiosis
67	European	33	0	*RECQL4*	NM_004260.4:c.1155delG:p.Glu388SerfsTer18	Het	3	DNA Repair and Meiosis
				*ATM*	NM_000051:c.3925G>A:p.Ala1309Thr/c.5558A>T:p.Asp1853Val	Pres Comp Het		DNA Repair and Meiosis
6	European	29	0	*GDF9*	NM_005260.5:c.261C>G:p.His87Gln	Het	3	Follicular Growth
8	European	33	0	*ATM*	NM_000051.4:c.8041G>C:p.Val2681Leu	Het	3	DNA Repair and Meiosis
12	European	35	0	*HSD17B4*	NM_000414.4: c.1165G>A: p.Gly389Arg	Het	3	Metabolism and Mitochondria
				*AIRE*	NM_000383.4:c.70A>G:p.Ser24Gly	Het		Follicular Growth
22	North-African	34	0	*POF1B*	NM_024921.4:c.986G>A:p.Arg329Gln	Het	3	Follicular Growth
23	European	31	0	*ERCC6*	NM_000124.4:c.4309T>A:p.Phe1437Ile	Het	3	DNA Repair and Meiosis
25	European	30	1	*BMPR1B*	NM_001256793.2:c.41T>G:p.Phe14Cys	Het	3	Follicular Growth
28	North-African	34	0	*WRN*	NM_000553.6:c.1909C>T:p.Arg637Trp	Het	3	DNA Repair and Meiosis
30	North-African	32	0	*BMPR2*	NM_001204.7:c.2233C>T:p.Leu745Phe	Het	3	Follicular Growth
				*EIF2B4*	NM_172195.3:c.259G>A:p.Ala87Thr	Het	3	Metabolism and Mitochondria
37	European	33	0	*BMPR2*	NM_001204.7:c.1862C>A: p.Thr621Lys	Het	3	Follicular Growth
38	European	34	0	*AMH*	NM_000479.5:c.872T>C:p.Leu291Pro	Het	3	Follicular Growth
39	North-African	32	0	*INHA*	NM_002191.4:c.338A>G:p.Tyr113Cys	Het	3	Follicular Growth
				*WDR62*	NM_001083961.2:c.3514C>T:p.Pro1172Ser	Het		DNA Repair and Meiosis
				*RCBTB1*	NM_018191.4:c.343G>C:p.Gly115Arg	Het		Follicular Growth
41	European	27	0	*CSMD1*	NM_033225.6:c.2521G>A:p.Gly841Arg	Het	3	Follicular Growth
46	European	27	0	*CSMD1*	NM_033225:c.8047C>G:p.P2683A	Het	3	Follicular Growth
59	European	33	0	*AR*	NM_000044.6:c.53C>G:p.Thr18Ser/c.1886-4A>G:p.?	Pres Comp Het	3	Ovarian Development
57	European	29	0	*FOXO3*	NM_001455.4:c.451T>G:p.Ser151Ala	Het	3	Follicular Growth
62	European	34	0	*ESR1*	NM_001122740.2:c.172G>T:p.Ala58Ser	Het	3	Ovarian Development
68	North-African	34	1	*STAR*	NM_000349.3:c.178G>A:p.Gly60Ser	Het	3	Metabolism and Mitochondria
69	North-African	31	0	*WDR62*	NM_001083961:c.3688C>T:p.Arg1230Cys	Het		DNA Repair and Meiosis
				*CSMD1*	NM_033225:c.7960C>T:p.Leu2654Phe	Het	3	Follicular Growth
71	North-African	35	0	*FANCM*	NM_020937.4:c.4865_4867delAAG:p.Glu1622del/c.1397-4A>G/c.5067G>A:p.Ala1689Ala	Pres Comp Het	3	DNA Repair and Meiosis
82	European	30	1	*LONP1*	NM_004793.4:c.2560C>T	Het	3	Metabolism and Mitochondria
88	European	29	0	*PCCB*	NM_000532:c.774C>G:p.His258Gln	Hom	3	Metabolism and Mitochondria
62	European	34	0	*GNAS*	NM_080425.3:c.2207A>G:p.Gln736Arg	Het	3	Follicular Growth

ACMG: classification of the variant according to the guidelines of the American college of Medical genetics. Het: heterozygous. Comp Het: compound heterozygous. Hom: homozygous. Pres Comp Het: presumed compound heterozygous.

## Data Availability

Original data generated and analyzed during this study are included in this published article or in the data repositories listed in the References.
